# Supplementary material for: Anti-Psoriatic Effect of Rheum palmatum L. and Its Underlying Molecular Mechanisms
Source: Int J Mol Sci. 2022 Dec 15;23(24):16000. doi: 10.3390/ijms232416000 (PMC9781959; doi:10.3390/ijms232416000)
Supplement: Supplementary file 1 [file ijms-23-16000-s001.zip › ijms-2037770-supplementary.pdf]

Table S1. Targets of RPE components.

Gene name	Protein name	Compounds
ACTA2	Actin, aortic smooth muscle	Emodin
ALR	Aldose reductase	Aloe-emodin, Rhein
BAX	Apoptosis regulator BAX	Aloe-emodin
BTK	Tyrosine-protein kinase BTK	Emodin
CALM	Calmodulin	Chrysophanol, Emodin, Physcion
CASP3	Caspase-3	Aloe-emodin, Emodin
CCNB1	G2/mitotic-specific cyclin-B1	Aloe-emodin
CDC2A	Cell division control protein 2 homolog	Aloe-emodin
CDKN1A	Cyclin-dependent kinase inhibitor 1A	Aloe-emodin, Emodin
CYP1A1	Cytochrome P450 1A1	Emodin
EGF	Pro-epidermal growth factor	Emodin
EIF6	Eukaryotic translation initiation factor 6	Aloe-emodin
F10	Coagulation factor Xa	Emodin, Physcion
F7	Coagulation factor VII	Emodin, Physcion
FASN	Fatty acid synthase	Aloe-emodin
GABRA1	Gamma-aminobutyric acid receptor subunit alpha-1	Chrysophanol
GM-CSF	Granulocyte-macrophage colony-stimulating factor	Emodin
HSP90AA1	Heat shock protein HSP 90	Aloe-emodin, Chrysophanol, Emodin, Physcion, Rhein
IGHG1	Ig gamma-1 chain C region	Aloe-emodin, Chrysophanol, Emodin, Physcion
IL1B	Interleukin-1 beta	Aloe-emodin, Emodin
JUN	Transcription factor AP-1	Rhein
MAOB	Amine oxidase [flavin-containing] B	Emodin
MMP1	Interstitial collagenase	Emodin
MMP9	Matrix metalloproteinase-9	Emodin

MYC	Myc proto-oncogene protein	Aloe-emodin, Emodin
NCOA1	Nuclear receptor coactivator 1	Emodin, Physcion
NCOA2	Nuclear receptor coactivator 2	Aloe-emodin, Chrysophanol, Emodin, Physcion, Rhein
NOS3	Nitric-oxide synthase, endothelial	Physcion
PCNA	Proliferating cell nuclear antigen	Aloe-emodin
PDE3A	CGMP-inhibited 3',5'-cyclic phosphodiesterase A	Chrysophanol
PIK3CG	Phosphatidylinositol-4,5-bisphosphate 3-kinase catalytic subunit, gamma isoform	Aloe-emodin, Chrysophanol, Emodin, Physcion, Rhein
PKIA	cAMP-dependent protein kinase inhibitor alpha	Aloe-emodin, Chrysophanol, Physcion
PPARG	Peroxisome proliferator-activated receptor gamma	Emodin
PRKACA	mRNA of PKA Catalytic Subunit C-alpha	Aloe-emodin, Chrysophanol, Emodin, Physcion
PRKCA	Protein kinase C alpha type	Aloe-emodin
PRKCD	Protein kinase C delta type	Aloe-emodin, Emodin
PRKCE	Protein kinase C epsilon type	Aloe-emodin, Emodin
PTGS1	Prostaglandin G/H synthase 1	Aloe-emodin, Chrysophanol, Emodin, Physcion, Rhein
PTGS2	Prostaglandin G/H synthase 2	Aloe-emodin, Chrysophanol, Emodin, Physcion, Rhein
RXRB	Retinoic acid receptor RXR-alpha	Physcion
SCN5A	Sodium channel protein type 5 subunit alpha	Chrysophanol, Physcion
SLC2A1	Solute carrier family 2, facilitated glucose transporter member 1	Emodin
SLC2A4	Solute carrier family 2, facilitated glucose transporter member 4	Emodin
TGFB1	Transforming growth factor beta-1	Emodin
TNF	Tumor necrosis factor	Aloe-emodin, Emodin
TOP2A	DNA topoisomerase II	Emodin, Physcion
TP53	Cellular tumor antigen p53	Aloe-emodin, Emodin
VEGFR1	Vascular endothelial growth factor receptor 1	Emodin
VEGFR2	Vascular endothelial growth factor receptor 2	Emodin
VEGFR3	Vascular endothelial growth factor receptor 3	Emodin

Table S2. GO functional enrichment analysis on potential targets of RPE components.

Category	GO term	Related genes
Biological Process	Positive regulation of pri-miRNA transcription by RNA polymerase II	*JUN*, *PPARG*, *TGFB1*, *TNF*, *TP53*
	Cellular response to cytokine stimulus	*CASP3*, *CDKN1A*, *HSP90AA1*, *IL1B*, *MMP9*, *PTGS2*, *TGFB1*, *TNF*, *TP53*
	Positive regulation of cellular metabolic process	*CCNB1*, *CDKN1A*, *EGF*, *PPARG*, *TGFB1*, *TP53*
	Regulation of pri-miRNA transcription by RNA polymerase II	*JUN*, *PPARG*, *TGFB1*, *TNF*, *TP53*
	Regulation of DNA binding	*EGF*, *JUN*, *MMP9*, *PPARG*, *TGFB1*
	Positive regulation of protein phosphorylation	*CDKN1A*, *EGF*, *HSP90AA1*, *IL1B*, *MMP9*, *TGFB1*, *TNF*, *TP53*
	Regulation of neuroinflammatory response	*IL1B*, *MMP9*, *PTGS2*, *TNF*
	Cytokine-mediated signaling pathway	*CASP3*, *CDKN1A*, *HSP90AA1*, *IL1B*, *MMP9*, *PTGS2*, *TGFB1*, *TNF*, *TP53*
	Regulation of fever generation	*IL1B*, *PTGS2*, *TNF*
	Positive regulation of heat generation	*IL1B*, *PTGS2*, *TNF*
Cellular Component	Cyclin-dependent protein kinase holoenzyme complex	*CCNB1*, *CDKN1A*
	Serine/threonine protein kinase complex	*CCNB1*, *CDKN1A*
	Intracellular organelle lumen	*CCNB1*, *HSP90AA1*, *MMP9*, *PTGS2*, *TGFB1*
	Platelet alpha granule lumen	*EGF*, *TGFB1*
	Actin-based cell projection	*ACTA2*, *TGFB1*
	Platelet alpha granule	*EGF*, *TGFB1*
	Secretory granule lumen	*EGF*, *HSP90AA1*, *TGFB1*
	Phosphatidylinositol 3-kinase complex, class I	*PIK3CG*
	Intracellular membrane-bounded organelle	*CASP3*, *CCNB1*, *CDKN1A*, *JUN*, *HSP90AA1*, *NOS3*, *PCNA*, *PPARG*, *PTGS1*, *TGFB1*, *TP53*
	Nucleus	*CASP3*, *CCNB1*, *CDKN1A*, *JUN*, *HSP90AA1*, *NOS3*, *PCNA*, *PPARG*, *TGFB1*, *TP53*

	Ubiquitin-like protein ligase binding	*CCNB1*, *CDKN1A*, *JUN*, *HSP90AA1*, *TP53*
	Protein tyrosine kinase binding	*HSP90AA1*, *PCNA*, *TP53*
	Transcription regulatory region nucleic acid binding	*JUN*, *PPARG*, *TNF*, *TP53*
	Protein kinase binding	*ACTA2*, *CCNB1*, *CDKN1A*, *HSP90AA1*, *TP53*
Molecular Function	Ubiquitin protein ligase binding	*CDKN1A*, *HSP90AA1*, *JUN*, *TP53*
	DNA polymerase binding	*HSP90AA1*, *PCNA*
	Histone acetyltransferase binding	*PCNA*, *TP53*
	Receptor ligand activity	*EGF*, *IL1B*, *TGFB1*, *TNF*
	Disordered domain specific binding	*HSP90AA1*, *TP53*
	Cytokine activity	*IL1B*, *TGFB1*, *TNF*

Table S3. KEGG enrichment analysis on potential targets of RPE components.

**KEGG pathway**	**Related genes**
IL-17 signaling pathway	*CASP3*, *HSP90AA1*, *IL1B*, *JUN*, *MMP9*, *PTGS2*, *TNF*
TNF signaling pathway	*CASP3*, *IL1B*, *JUN*, *MMP9*, *PTGS2*, *TNF*
MAPK signaling pathway	*CASP3*, *EGF*, *IL1B*, *JUN*, *TGFB1*, *TNF*, *TP53*
Cell cycle	*CCNB1*, *CDKN1A*, *PCNA*, *TGFB1*, *TP53*
Th17 differentiation	*HSP90AA1*, *IL1B*, *JUN*, *TGFB1*
NF-kappa B signaling pathway	*IL1B*, *PTGS2*, *TNF*
VEGF signaling	*NOS3*, *PTGS2*
Cytokine-cytokine receptor interaction	*IL1B*, *TGFB1*, *TNF*
TGF-beta signaling pathway	*TGFB1*, *TNF*
JAK-STAT signaling pathway	*CDKN1A*, *EGF*
